# TXNIP interaction with the Her-1/2 pathway contributes to overall survival in breast cancer

**DOI:** 10.18632/oncotarget.3096

**Published:** 2014-12-30

**Authors:** Weiwei Nie, Weisun Huang, Wenwen Zhang, Jing Xu, Wei Song, Yanru Wang, Aiyu Zhu, Jiayan Luo, Guichun Huang, Yucai Wang, Xiaoxiang Guan

**Affiliations:** ^1^ Department of Medical Oncology, Jinling Hospital, School of Medicine, Southern Medical University, Guangzhou, P.R. China; ^2^ Department of Medical Oncology, Jinling Hospital, Medical School of Nanjing University, Nanjing, P.R. China; ^3^ Department of Medicine, Rutgers New Jersey Medical School, Newark, NJ, USA

**Keywords:** TXNIP, p27, Her-1/2 inhibitor, breast cancer

## Abstract

Previous studies have indicated that Her-2 induction causes a strong decrease in thioredoxin interaction protein (TXNIP) in breast cancer cells. However, little is known regarding the prognostic value of TXNIP in clinical breast cancer patients with anti-Her-2 treatment. Using a tissue microarray, we detected TXNIP and p27 expression in breast cancer tissue, as well as corresponding noncancerous tissues. We found that TXNIP expression was associated with better overall survival (OS) in these 150 breast cancer patients and that TXNIP and Her-2 expression status were significantly inversely correlated (r=-0.334, *P*<0.001). These results were validated in another 101 breast cancer tissue samples (r=-0.422, *P*<0.001). Moreover, TXNIP expression increased significantly following treatment of the human breast cancer cell lines BT474 and SK-BR-3 with a Her-1/2 inhibitor. Furthermore, TXNIP transfection induced p27 expression and G_1_ cell cycle arrest and apoptosis. Taken together, our findings suggest that TXNIP plays a critical role in anti-Her-1/Her-2 treatment and may be a potential prognostic marker in breast cancer.

## INTRODUCTION

As a negative regulator of thioredoxin (TRX), thioredoxin interaction protein (TXNIP) (also known as Vitamin D3 up-regulated protein 1 (VDUP-1) or thioredoxin binding protein 2 (TBP-2)) has strong growth suppressive, metastasis inhibitory and proapoptotic functions [1]. Subsequently, it has been identified as a tumor suppressor gene in various solid tumors and hematological malignancies, including breast cancer [2]. A recent study on the association of TXNIP expression and metastasis-free survival of breast cancer patients found that TXNIP was associated with better prognosis [2]. They induced Her-2 in MCF-7 cells, which strongly inhibited TXNIP expression, following a strong increase reactive oxygen species levels. This Her-2-dependent repression of TXNIP expression was interpreted as being part of Her-2-triggered survival program.

It has also been reported that the redox balance in cancer cells is disrupted by oxidative stress caused by accelerated cell proliferation, constant stimulation of growth promoting signaling pathways and alterations in metabolic activity [3]. Therefore, as a major redox regulator, TXNIP has recently been proposed as a therapeutic target for cancer treatment [4].

TXNIP may also be a prognostic marker of breast cancer response to anthracycline-based chemotherapy [5]. Based on immunohistochemical (IHC) staining of 98 locally advanced primary breast cancer patients, TXNIP was found to be an independent prognostic factor for distant metastasis-free survival [5]. However, despite the ongoing evaluation of TXNIP as a therapeutic target, little is known about the prognostic value of TXNIP in breast cancer patients receiving anti-Her-2 treatment and its relevance to overall survival (OS). Here, we investigated TXNIP expression in early stage breast cancer patients and its association with OS. We further evaluated TXNIP expression in response to anti-Her-2 treatment, to examine the role of TXNIP in the Her-1/2 pathway as well as its effects on OS of breast cancer patients.

## RESULTS

### TXNIP and p27 are associated with OS in breast cancer patients

**Figure 1:**
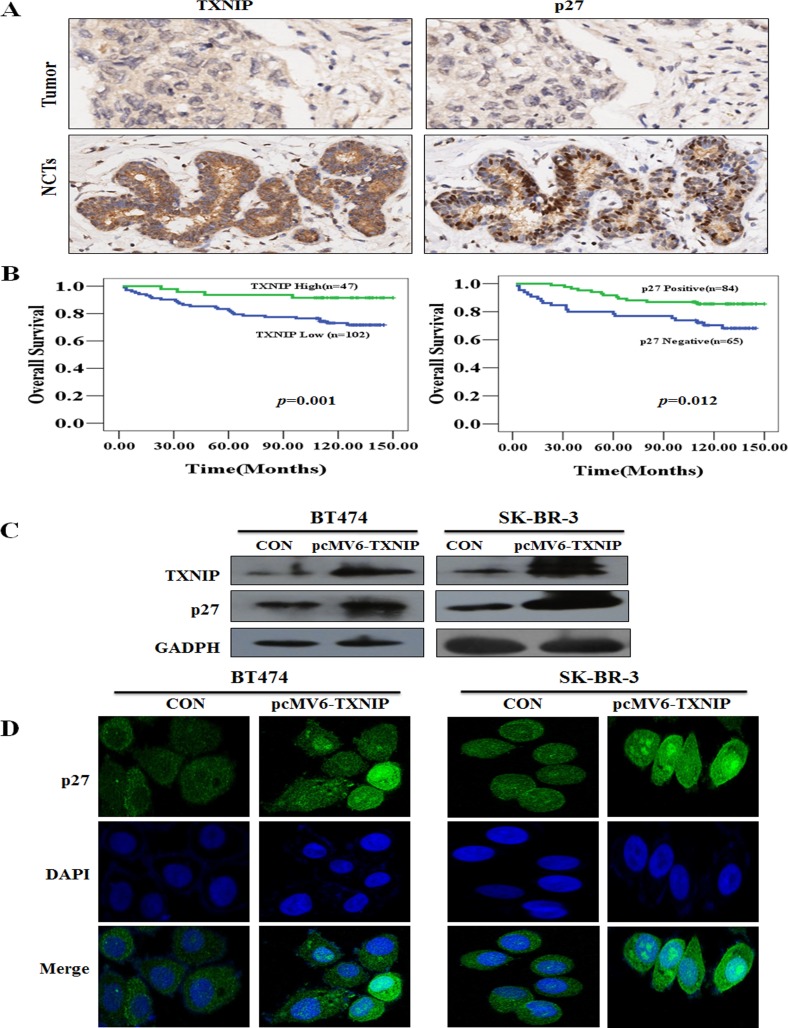
The association of TXNIP and p27 with OS in breast cancer tissues and NCTs in a tissue-array (A) Low expression of TXNIP and negative expression of p27 in breast cancers compared with NCTs were demonstrated by IHC of breast cancer tissues and NCTs. (B) High TXNIP or positive p27 expression was associated with longer OS (*P* =0.001, *P* =0.012, respectively). (C) TXNIP and p27 expressions were evaluated by western blotting 48 h after transfection with the TXNIP overexpression plasmid. (D) p27 localization and expression were determined by fluorescent microscopy after transfection with TXNIP overexpression plasmid. Nuclei were stained with DAPI.

To evaluate the putative association between TXNIP and p27 with OS, we performed IHC staining against these proteins in malignant tumor and corresponding noncancerous tissues (NCTs) samples from 150 patients using a tissue microarray (Figure 1A). We found that high TXNIP or p27 expression was associated with better OS (Figure 1B, *P*=0.001 and *P*=0.012). Demographic, pathological and clinical variables were collected and correlations of TXNIP expression with clinicopathological factors of breast cancer patients were determined (Table 1). Of the 150 tumor tissues, 48 cases (32%) and 102 cases (68%) expressed TXNIP at high and low levels, respectively. TXNIP and p27 expressions were decreased in breast cancer tissue compared with NCTs (*P* =0.003, *P* <0.001, respectively) (Table 2). We next analyzed the correlation between TXNIP, p27 and Her-2 expression at the tumor microarray or at tumor tissues, respectively. A significant negative correlation was found between TXNIP and Her-2 status using the breast cancer tissue-array (n=150) (r=-0.334, *P*<0.001), which was again validated in the external cohort (n=101) (r=-0.422, *P*<0.001) (Table 3). Similarly, a significant negative correlation was found between p27 and Her-2 status using the breast cancer tissue-array (n=150) (r=-0.344, *P*<0.001) that was confirmed in the external cohort (n=101) (r=-0.284, *P*=0.004) (Table 4). Therefore, TXNIP and p27 are inversely associated with Her-2 status. Accordingly, a significant positive correlation was found between TXNIP and p27 using the breast cancer tissue-array (n=150) (r=0.340, *P*<0.001) that was confirmed in the external cohort (n=101) (r=0.331, *P*=0.001) (Table 5). SK-BR-3 and BT474 cell lines were transfected with pcMV6-TXNIP plasmid to determine whether TXNIP contributes to p27 expression, followed by western blotting and confocal microscopy to detect p27 and TXNIP expression. We found that pcMV6-TXNIP-transfection enhanced p27 expression in BT474 and SK-BR-3 cells (Figure 1C and D).

**Table 1:** Relationship between expression of TXNIP and clinicopathologic characteristics of breast cancer patients

Variables	TXNIP high (n=48)		TXNIP low (n=102)		*P*-value	Long-rank test (*P*)
	No.	%	No.	%		
Age					0.155	0.089
≤53 years	33	68.8	57	55.9		
>53 years	15	31.2	45	44.1		
Histological^a^					0.688	< 0.001
I	4	9.1	11	11.3		
II-III	40	90.9	86	88.7		
TNM stage^b^					0.025	< 0.001
I-II	39	83.0	65	65.0		
III-IV	8	17.0	35	35.0		
ER status^c^					0.610	0.003
negative	12	27.9	30	32.3		
positive	31	72.1	63	67.7		
PR status^d^					0.420	0.029
negative	15	34.1	38	41.3		
positive	29	65.9	54	58.7		

Note:

a 4 and 5 samples of the histological status of TXNIP high and low expression group were missed respectively.

b 1 and 2 samples of the TNM stage of TXNIP high and low expression group were missed respectively.

c 5 and 9 samples of ER status of TXNIP high and low expression group were missed respectively.

d 4 and 10 samples of PR status of TXNIP high and low expression group were missed respectively

**Table 2:** TXNIP and p27 expression in breast cancers and NCTs microarray

	Breast cancers (n=150)		NCTs (n=90)		*P* value
	No.	%	No.	%	
TXNIP					0.003
0	70	46.7	20	22.2	
+	32	21.3	5	5.6	
++	40	26.7	40	44.4	
+++	8	5.3	25	27.8	
p27					< 0.001
0	65	43.3	9	10.0	
+	45	30.0	17	18.9	
++	28	18.7	35	38.9	
+++	12	8.0	29	32.2	

Note: NCTs: corresponding noncancerous tissues. TXNIP or p27 expression between breast cancer and NCTs was tested by Two-Related-samples test.

**Table 3:** Correlative analysis of the TXNIP expression with Her-2 at tumor microarray and tumor tissues

	Testing Set		Validation Set	
	Tumor microarray (n=150)		Tumor tissues (n=101)	
	Her-2 (positive)	Her-2(negative)	Her-2(positive)	Her-2 (negative)
TXNIP(high)	7	41	4	58
TXNIP(low)^a^	43	46	16	23
*r*	**-0.334**		**-0.422**	
*P*	**< 0.001**		**< 0.001**	

Note:

a 13 samples of Her-2 status in TXNIP low expression group of tumor microarray were missed.

**Table 4:** Correlative analysis of the p27expression with Her-2 at tumor microarray and tumor tissues

	Testing Set		Validation Set	
	Tumor microarray (n=150)		Tumor tissues (n=101)	
	Her-2 (positive)	Her-2(negative)	Her-2(positive)	Her-2 (negative)
p27(positive)^a^	18	62	4	45
p27(negative)^b^	32	25	16	36
*r*	**-0.344**		**-0.284**	
*P*	**< 0.001**		**0.004**	

Note:

a 5 samples of Her-2 status in p27 positive group of tumor microarray were missed.

b 8 samples of Her-2 status in p27 negative group of tumor microarray were missed.

**Table 5:** Correlative analysis of the TXNIP and p27expression at tumor microarray and tumor tissues

	Testing Set		Validation Set	
	Tumor microarray (n=150)		Tumor tissues (n=101)	
	TXNIP (high)	TXNIP(low)	TXNIP(high)	TXNIP (low)
p27(positive)	39	46	26	23
p27(negative)	9	56	11	41
*r*	**0.340**		**0.331**	
*P*	**< 0.001**		**0.001**	

### TXNIP causes G_1_ cell cycle arrest and inhibits cell proliferation in Her-1/2 positive breast cancer cell lines

**Figure 2:**
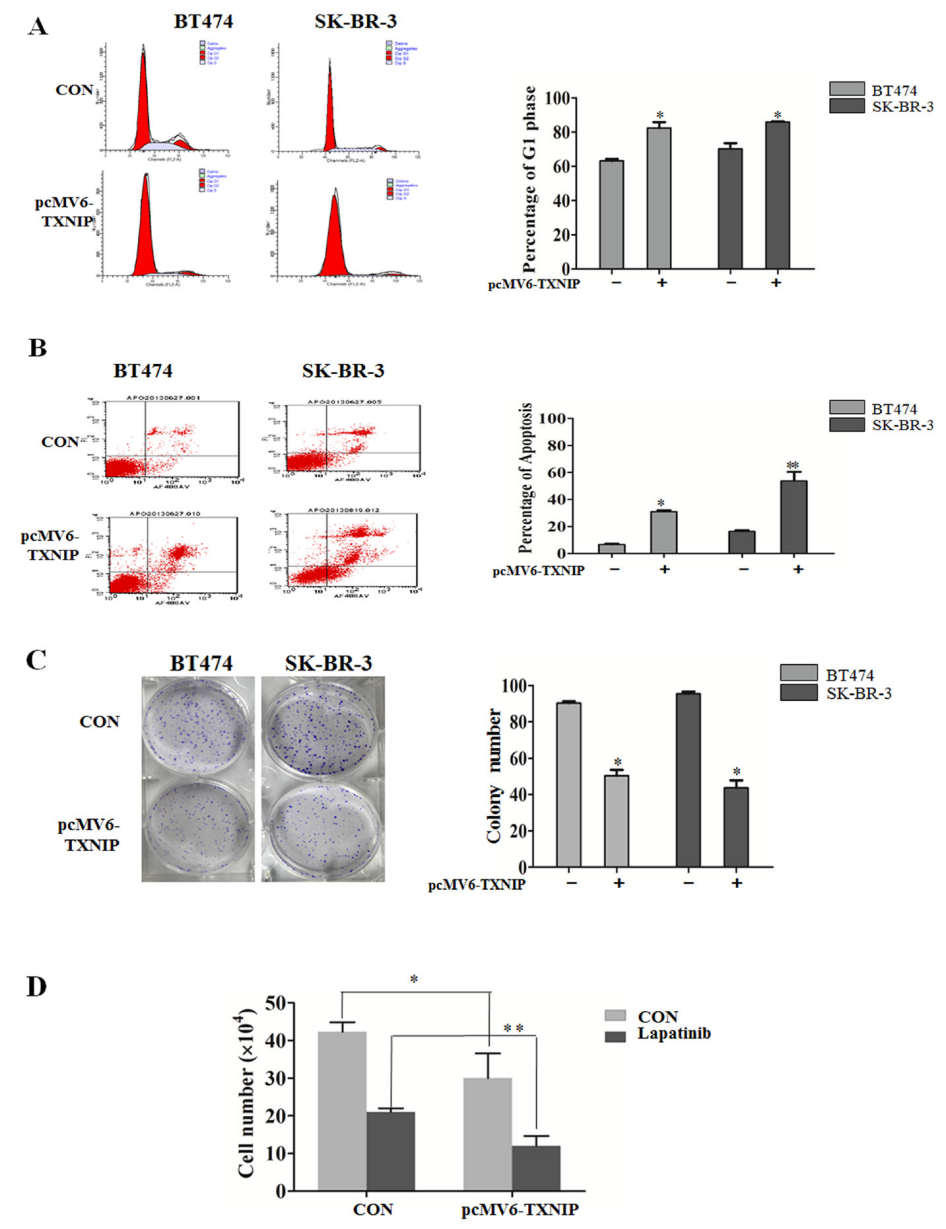
TXNIP causes G cell cycle arrest and inhibits cell proliferation in HER-1/2 positive breast cancer cell lines (A and B) The percentage of G_1_ phase arrest and apoptosis of BT474 and SK-BR-3 cells was determined via flow cytometry 48 h after transfection with the TXNIP overexpression plasmid. (C) The colony formation assay was used to measure the proliferating ability of BT474 and SK-BR-3 cells. (D) Transfected cells were treated with 0.5 μM lapatinib for 48 h, cell numbers were counted and cell viability was determined by trypan blue dye exclusion. *P < 0.05, ** *P* < 0.01.

Recent studies suggest that TXNIP plays an important role in suppressing cellular growth [6] and in inducing apoptosis [7]. In this study, we also demonstrated that overexpression of TXNIP could induce p27 expression in BT474 and SK-BR-3 cell lines. It was reported that p27 was a crucial negative regulator of the protein kinase CDK2/cyclin E and could cause G_0_/G_1_ cell cycle arrest [8]. Using flow cytometry to explore the role of TXNIP in the cell cycle control and cell proliferation in breast cancer, we found that TXNIP overexpression enhanced G_1_ cell cycle arrest (Figure 2A) and induced apoptosis both in BT474 and SK-BR-3 cell lines (Figure 2B). Moreover, upregualtion of TXNIP suppressed the proliferative ability of BT474 and SK-BR-3 cells (Figure 2C). We previously indicated that lapatinib, a dual Her-1/Her-2 tyrosine kinase inhibitor, has potent antitumor effects against human breast cancer [9]. To investigate whether TXNIP enhances lapatinib-induced inhibition of cell proliferation, we transiently transfected SK-BR-3 cells with a pcMV6-TXNIP-expressing vector, and treated them with 0.5μM lapatinib for 48 h. We found that TXNIP expression inhibited cell proliferation both in the presence and absence of lapatinib (Figure 2D). Taken together, our results suggest that TXNIP may enhance lapatinib-induced inhibition of cell proliferation in Her-1/2 positive breast cancer cell lines.

### TXNIP expression is regulated by Her-1/2 pathway inhibitors

**Figure 3:**
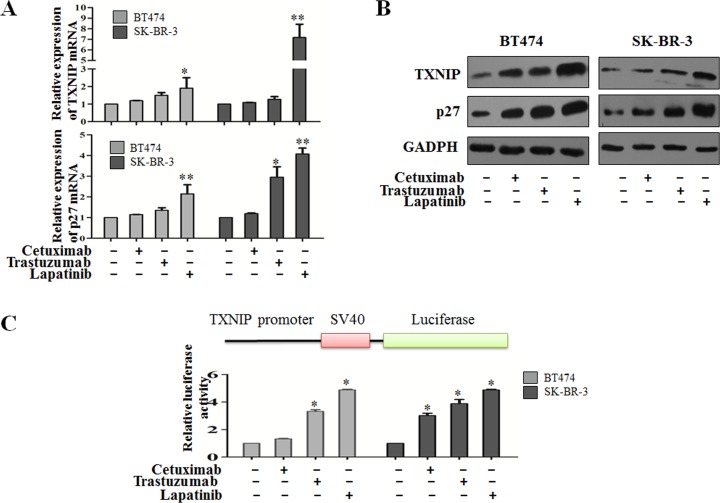
Her-1/2 pathway inhibitors regulate TXNIP protein and mRNA expression (A and B) After treatment with 10 μg/ml cetuximab, 20 μg/ml trastuzumab or 0.5 μM lapatinib, TXNIP and p27 protein and mRNA expression were determined by western blotting and qRT-PCR. (C) A firefly luciferase reporter containing the TXNIP promoter sequence was transfected into BT474 and SK-BR-3 cells along with 10 μg/ml cetuximab, 20 μg/ml trastuzumab or 0.5 μM lapatinib. Cells were assayed 24 h after transfection, using a luciferase assay kit. Results were expressed relative to the luciferase activity in control cells (which was set to 1). The results are presented as the mean ± S.D. *P < 0.05, **P < 0.01.

To investigate whether the Her-1/2 pathway affects TXNIP expression in human breast cancer cells, BT474 and SK-BR-3 cells were treated with 10 μg/ml cetuximab, 20 μg/ml trastuzumab or 0.5 μM lapatinib. TXNIP and p27 mRNA levels were detected by RT-PCR and protein levels by western blot analysis. As shown in Figure 3A and B, cetuximab, trastuzumab and lapatinib treatment induced TXNIP and p27 expression in BT474 and SK-BR-3 cells at varying levels with lapatinib causing the most significantly upregulation of TXNIP and p27 expression.

To study the regulatory mechanism of Her-1/2-dependent inhibition of TXNIP, luciferase activity was assayed after transfecting BT474 and SK-BR-3 cells with a TXNIP-promoter plasmid. Twenty-four hours after TXNIP promoter plasmid transfection the relative luciferase activities were enhanced in all three treated groups. TXNIP expression could thus be induced by transcriptional inhibition of Her-1/2 (Figure 3C). Collectively, our results show that TXNIP transcription is upregulated by inhibition of Her-1/2 signaling.

## DISCUSSION

In this study, we found a significant negative correlation between TXNIP and Her-2 status in breast cancer, and showed that inhibition of the Her-1/2 pathway resulted in increased TXNIP expressions. These findings are consistent with previous reports indicating that Her-2 induction strongly inhibits TXNIP expression in breast cancer cells [2]. Also, using a tissue array, we found decreased expression of TXNIP and p27 in breast cancer tissue, compared with NCTs. Further study indicated that overexpression of TXNIP was accompanied by increased p27 expression, which resulted in G_1_ arrest and inhibition of cell proliferation. This may explain our finding that high TXNIP expression was associated with increased OS, because p27, a cyclin-dependent kinase inhibitor,plays a pivotal role in inhibiting cell proliferation and apoptosis [10]. Moreover several studies­ (including a meta-analysis conducted by us) have demonstrated that reduced p27 is an independent prognostic factor for poor overall and disease-free cancer survival [11].

The human EGFR family comprises four closely related transmembrane glycoprotein receptors that contain an extracellular ligand binding domain and an intracellular receptor tyrosine kinase domain. This family includes Her-1 (also known as ErbB1 and EGFR), Her-2 (also known as HER-2/neu and ErbB2), Her-3 (also known as ErbB3), and Her-4 (also known as ErbB4) [1]. Treatment of breast cancer cells with a combination of Her-1 and Her-2 inhibitors results in a synergistic antitumor effect mediated by the modulation of several downstream signaling proteins and cell cycle regulatory proteins, including transcriptional and posttranscriptional regulation of p27 expression [12]. As previously observed, the dual Her-1/2 inhibitor lapatinib regulates p27 expression at the transcriptional as well as the post-translational level [9]. Meanwhile, we showed that cetuximab, trastuzumab and lapatinib treatment led to increased TXNIP and p27 expression in BT474 and SK-BR-3 cells. Taken together, TXNIP may therefore potentially impact cell survival through its interaction with p27 in the Her-1/2 pathway.

To our knowledge, few study has been reported a direct influence of Her-1/2 signaling on TXNIP mRNA expression. Glucose induces TXNIP expression,and ROS triggers the dissociation of TXNIP from thioredoxin, leading to increased TXNIP availability for activation of the NLRP3 inflammasome [13]. Also, hyperglycemia regulates thioredoxin-ROS activity through induction of TXNIP in breast cancer derived cells [14, 15]. Inhibition of EGF signaling in Her-2-positive breast cancer has been associated with glucose deprivation and energetic stress [16]. Accordingly, treatment with lapatinib is associated with increased expression of glucose deprivation response network genes [17]. Therefore, TXNIP may be induced via hyperglycemia regulated thioredoxin-ROS activity in Her-1/2 signaling pathway. Information regarding this mechanism will require further investigation in the future.

**Figure 4:**
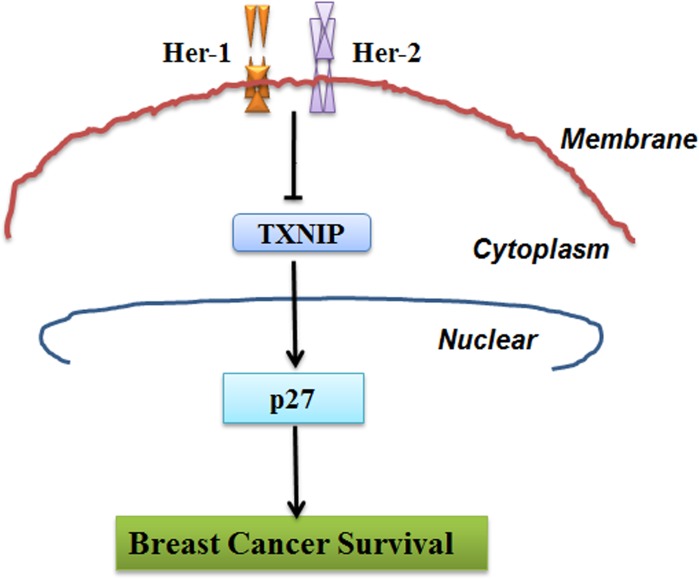
Schematic model demonstrating the critical role of TXNIP and p27 in Her-1/2 mediated cell proliferation in breast cancer cell

Collectively, our results suggest that dynamic regulation of TXNIP interaction with the Her-1/2 pathway contributes to OS of breast cancer (Figure 4). When treated with the anti-Her-1 monoclonal antibody cetuximab, the anti-Her-2 monoclonal antibody trastuzumab or the dual Her-1/2 inhibitor lapatinib, TXNIP mRNA and protein levels were increased in breast cancer cells. Overexpression of TXNIP was accompanied by increased p27 expression, resulting in G_1_ phase arrest and inhibition of cell proliferation. Moreover, high TXNIP and p27 expression were associated with better OS in human breast cancer patients. In conclusion, TXNIP may interrupt Her receptor family mediated oncogenic pathways thereby increasing OS in breast cancer patients. Our findings suggest TXNIP plays a critical role in anti-Her-1/Her-2 treatment and may be a potential prognostic marker in breast cancer.

## MATERIALS AND METHODS

### Clinical samples

Breast cancer tissues and corresponding noncancerous tissues (NCTs) sections containing HBre-Duc170Sur-01 (170 cancer cases) and HBre-Duc090Sur-01 (90 NCTs) were provided by Outdo Biotech (Shanghai, China). The validation set containing 101 breast tumor tissues were collected from Wuxi City People's Hospital (Jiangsu, China) between January 2012 and December 2013. None of the patients received chemotherapy or radiotherapy prior to surgery. The experiments were approved by the Ethics Committee of Jinling Hospital and were conducted in compliance with the Helsinki Declaration. Disease histology was determined in accordance with the criteria of the World Health Organization. Pathologic staging was performed in accordance with the current International Union against Cancer tumor-lymph node metastasis classification.

### Immunohistochemistry

Breast tumor tissue samples were deparaffinized in xylene. Heat-mediated antigen retrieval was applied using citrate buffer (BioGenex Laboratories, San Ramon, CA). Antibody staining was visualized with DAB (Sigma, D-5637) and hematoxylin counterstain. The H-score method was used in this trial. We multiplied the percentage score by the staining intensity score. The percentage of positively stained cells was scored as “−” (0%), “+” (1%-25%), “++” (26%-50%) or “+++” (51%-100%). Intensity was scored as “−” (negative), “+” (weak), “++” (moderate) and “+++” (strong). Immunohistochemical scoring was performed without prior knowledge of the clinical response. Immunostained sections were scanned using a microscope (Aiovert 200; Carl Zeiss).

### Cell cultures and Her-1/2 inhibitor treatment

Breast cancer cell lines (BT474 and SK-BR-3) were purchased from the American Type Culture Collection (Manassas, VA, USA) and cultured in RPMI1640 medium (GIBCO, Gaithersburg, MD, USA) supplemented with 10% fetal bovine serum (FBS) and 1% penicillin/streptomycin at 37°C in a humidified atmosphere with 5% CO_2_. Cetuximab (Erbitux; MerckKGaA, Darmstadt, Germany) and trastuzumab (Herceptin, F. Hoffmann-La Roche, Basel, Switzerland) were dissolved in sterile apyrogen water and stored at 4°C. Lapatinib (Tykerb, GlaxoSmithKline, Research Triangle Park, NJ, USA) was dissolved in dimethyl sulfoxide (dimethyl sulfoxide as a stock solution at 10mM) and stored at −20°C. BT474 and SK-BR-3 cells were treated with cetuximab and trastuzumab at a final concentration of 10 μg/ml and 20 μg/ml in the culture medium, respectively. BT474 and SK-BR-3 cells were treated with lapatinib at a final concentration of 0.5 μM in the culture medium.

### Plasmids and transient transfection

The pcMV6-TXNIP plasmid was a gift from Xiaofeng Le (MD Anderson Cancer Center). The pGL3-TXNIP-promoter plasmid was synthesized by GENEray Biotechnology (Shanghai, China) and the promoter sequence was: 3′-GGAGGCTCGTGCTGCCCTCGTGCA CATCCCTCCCATTGGCTGCCCGGTCCTTGTTTACC AGGAGCCCGACCAATCAGTGAGATCGCTGTGGCG CGTGGACACGGTGTGCTCCTGGCTGGGAAAATGG TTGTTGCGCTCTGGAGCGGCGCAGGGAGGGGGGAAGGAGAGGAAGGAGAGGAAGG AGGGGAAGGAGGGGGCTGTTGAGCGTCTTCTCCCGGGTCCAGTGGAAGGAGGATCCCACTGACCCTAAAACCTAGCCAGG-5′. A random sequence was used as negative control. BT474 and SK-BR-3 cells were plated in six-well plates at a density of 1×10^6^ cells/plate 24 h prior to transfection. Cells were transfected with either the TXNIP overexpression sequence or the random sequence using TurboFect Transfection Reagent (Thermo Scientific) according to the manufacturer's protocol. After incubation at 37°C for 48 h, cells were collected to measure TXNIP and p27 expression by Western blot analysis.

### Western blotting

Total protein was extracted using RIPA buffer supplemented with protease and phosphatase inhibitors and quantitied using a BCA kit (Thermo Scientific, Pittsburgh PA, USA) and 20 μg of protein was loaded in each lane and separated on a sodium dodecylsulfate-polyacrylamide (SDS-PAGE) gel and blotted onto nitrocellulose. Blots were blocked with 5% dry milk in tris-buffered saline/0.1% tween-20 and incubated overnight with a diluted solution of primary antibody at 4°C, followed by horseradish peroxidase-conjugated secondary antibody (1:5000) for 2 h. The specific antibodies used for western blot were mouse anti-TXNIP antibody (1:100, MBL) and mouse anti-p27 antibody (1:1000, ab54563). Bands were normalized to GAPDH expression, which was used as an internal loading control. Results from at least two separate experiments were analyzed.

### Immunofluorescence analysis

Immunofluorescent staining was used to verify the expression and subcellular localization of p27. Cells were plated onto glass cover slips in six-well plates and transfected with pcMV6-TXNIP for 48 h. After washing with PBS and fixing in 4% paraformaldehyde for 20 min, cells were permeabilized with 0.1% Triton X-100 for 10 min and incubated for 1 h at 37°C with mouse anti-p27-antibody (1:200, ab54563). The cells were then washed with PBS and incubated for 30 min at 37°C with secondary anti-mouse IgG conjugated with FITC (Invitrogen; 1:200). Cells were then incubated with goat Texas red–conjugated anti-mouse IgG (Becton Dickinson-PharMingen) at a dilution of 1:200 in blocking buffer for 30 min followed by extensive washing with PBS. For all stains, cells were incubated with 5 μg/ml of 4, 6-diamidino-2-phenylindole (DAPI; Sigma) to stain the nuclei. Finally, cells were mounted with mounting solution (DAKO, Glostrup, Denmark) and examined under a LSM510 confocal microscope (Carl Zeiss, Gottingen, Germany).

### Cell cycle and apoptosis analysis

BT474 and SK-BR-3 cells were transfected with pcMV6-vector and pcMV6-TXNIP plasmid for 48 h, then harvested by trypsinization (not with EDTA) and washed with PBS. Analysis of the cell cycle and apoptosis was performed as previously described [18].

### Colony formation assay

Cells were seeded in six-well plates at a density of 500 cells per well. After 14 days, the colonies were fixed with 70% ethanol and stained with 0.1% crystal violet. Colonies larger than 1 mm were manually counted. These experiments were repeated at least three times.

### Cell proliferation assays

BT474 and SK-BR-3 cells were seeded on six-well plates (10^5^ cells/ well). After 24 h, cells were transfected with pcMV6-vector TXNIP. The effects of 0.5 μM lapatinib on cell proliferation were measured 48 h following initial exposure to treatments by counting cells, with cell viability determined by trypan blue dye exclusion.

### Dual-Luciferase Reporter Assay

Cells (1×10^5^/well in a six-well plate) were transiently transfected with 1 μg of luciferase construct (pGL3-promoter-luc, and pGL3-Luc) and 0.1 μg of pRL-Tk (Promega, Madison, USA) together with the indicated plasmids using Lipofectamine/plus reagent (Invitrogen, Carlsbad, CA, USA). After transfection for 24 h, the cells were harvested with the lysis buffer, and luciferase activities of cell extracts were measured by a luminometer (Centro XS3 LB960, Berthold, Germany) with the use of the Dual-luciferase assay system (Promega, Madison, USA) according to the manufacturer's instructions. Relative fire fly luciferase activity was normalized to Renilla luciferase activity and activity was expressed as fold induction. Each assay was performed in triplicate and the experiment was repeated at least three times.

### RNA isolation and quantitative RT-PCR

Total RNA was extracted from cultured cells using TRIzol Reagent (Invitrogen, Carlsbad, CA, USA) according to the manufacturer's instructions. For quantitative RT-PCR analysis of TXNIP and p27, 1μg total RNA was reverse transcribed to cDNA with oligdT primers and Thermoscript (TaKaRa, Dalian, China). Primer sequences (forward and reverse, respectively) were as follows: TXNIP, 5′-AGAGCCAACAGAACAGAAGAA-3′ and 5′-AGAGGCAGATCATTTAAGAGTG-3′; p27, 5′-AGAGCCAACAGAACAGAAGAA-3′ and 5′-AGAGGCAGATCATTTAAGAGTG-3′; β-actin, 5′-AGGGAAATCGTGCGTGAC-3′ and 5′-CGCTCATTGCCGATAGTG-3′. Real-time PCR analyses of TXNIP and p27 were performed on an ABI 7300 Sequence Detection System (Applied Biosystems, Foster City, CA, USA) using SYBR green dye (Invitrogen, Carlsbad, CA, USA). A 20 μl reaction volume included 1μl cDNA, 1× QuantiTect SYBR green PCR Master Mix, and 0.5 μM of sense and 0.5 μM of antisense primer. All PCRs were performed in triplicate. Threshold cycles (CT) were determined using fixed threshold settings.

### Statistical analyses

SPSS Statistics 16.0 (SPSS Inc.) was used for statistical analysis. Data were analyzed using one-way ANOVA or a Student's t-test. Data are presented as means ± the standard deviation (SD) of three independent experiments. The χ2 tests were used to compare the distribution of demographic variables between TXNIP high expression group and TXNIP low expression group. The log-rank test was used to assess statistical significance of Kaplan-Meier plots. For IHC data, the Two-Related-samples test between breast cancers and NCTs was used, and the statistical significance of the correlation between TXNIP expression level and Her-2 expression level in breast cancers or in NCTs was estimated by Spearman's Rank correlation analysis and statistical significance was again defined as *P < 0.05 or **P < 0.001.
